# Illness uncertainty, anxiety and depression in Chinese patients with glaucoma or cataract

**DOI:** 10.1038/s41598-018-29489-1

**Published:** 2018-08-03

**Authors:** Dandan Zhang, Zhigang Fan, Xinbo Gao, Wenmin Huang, Qiongman Yang, Zhongwen Li, Mingkai Lin, Huiming Xiao, Jian Ge

**Affiliations:** State Key Laboratory of Ophthalmology, Guangdong Provincial Key Laboratory of Ophthalmology and Visual Science, Zhongshan Ophthalmic Center, Sun Yat-sen University, Guangzhou, China

## Abstract

This study evaluated illness uncertainty, anxiety and depression among glaucoma patients and cataract patients in China. 263 patients with primary glaucoma and 100 patients with age-related cataract were recruited sequentially from Zhongshan Ophthalmic Center between October 2013 and March 2016. All the participants completed questionnaires for socio-demographic information, Mishel Uncertainty in Illness Scale (MUIS) and Hospital Anxiety and Depression Scale (HADS). 25 of the 263 glaucoma patients and 21 of the100 cataract patients finished two copies of the same questionnaires before and after surgery. Statistics were analyzed using SPSS17.0 software. We observed that glaucoma patients had higher MUIS and HADS score than did cataract patients. Multivariate logistic regression analysis indicated risk factors for illness uncertainty, anxiety and depression for glaucoma patients were high HADS score, poor visual acuity (VA) in the better eye and education level respectively. Risk factors for the same parameters of cataract patients were high HADS-A score, poor VA in the better eye and high illness uncertainty respectively. Scores of MUIS and HADS both decreased after surgery, but the change in HADS score among glaucoma patients was not significant. Clinical workers should take these factors into account to improve therapy, especially for glaucoma patients who undergo surgery.

## Introduction

Glaucoma and cataract are the two leading causes of blindness worldwide^1,2^. It is estimated that there will be 11.2 million cases of blindness worldwide due to glaucoma and more than a quarter of them live in China by 2020^3^. Meanwhile, 53 million people over 60 years of age will be blind by 2020 due to cataract^4^. The loss of vision is not only associated with physical suffering, but also with mental distress, including illness uncertainty and psychological disorders. Mental distress has always been neglected by doctors and nurses, which can negatively affect therapy and quality of life^5^.

The most common manifestations of psychological distress are anxiety and depression. Previous studies have evaluated anxiety and depression in many diseases, including glaucoma and cataract^6–10^. These studies show that patients with glaucoma or cataract have more serious anxiety and depression compared with healthy people. The fear of being blindness, heavy economic burden, quality of life deterioration due to restricted physical activities and inadequate communication or poor understanding of medical terms can cause anxiety and depression among patients. The potential impact factors that may contribute to such psychological distress were assessed, but the conclusion remains uncertain.

Once patients are diagnosed with glaucoma or cataract, the lack of communication and information stimulates the process of uncertainty, which includes unfamiliarity, ambiguity, vagueness, doubt and unpredictability^11^. Illness uncertainty is always present in chronic diseases and is associated with the psychological states of patients^12^. To the best of our knowledge, assessments of illness uncertainty have not been applied to glaucoma or cataract patients, and the relationship between illness uncertainty and psychological distress in glaucoma and cataract patients remains unknown.

The mood status of a patient is an important aspect of the overall health. The goal of the present study was to evaluate the condition of illness uncertainty and psychological disorders among glaucoma patients and cataract patients, and to assess the relationships between illness uncertainty, anxiety, depression and some potential impact factors, including surgery. The two scales applied in this study are MUIS and HADS assessing illness uncertainty and psychological disorders, respectively. Both the two scales are widely used in studies for various kinds of patients, and we believe they are suitable for our study.

## Results

### Demographic characteristics of the participants

The demographic and baseline characteristics of the study population are summarized in Table 1. The glaucoma group included 263(123 women) Chinese patients with a mean age of 57.20 ± 13.94 years (range, 18–90 years), and the cataract group consisted of 100 cataract subjects (53 women) with a mean age of 70.23 ± 9.78 years (range, 43–90 years). The two groups did not differ significantly (p > 0.05) in terms of sex, education level, medical insurance, VA in the worse eye and duration of diagnosis. However the differences in age, economic status and VA in the better eye were significant (p < 0.05).

**Table 1:** Demographic characteristics and scores of the study subjects.

Variables	Glaucoma	Cataract	*P*
N	263	100	
Age (y)	57.20 ± 13.94	70.23 ± 9.78	<0.001*
Sex, Female/Male	123/140	53/47	0.289
Education level			0.274
College or higher	42 (15.97%)	15 (15.00%)	
High school	124 (47.15%)	50 (50.00%)	
Primary or below	97 (36.88%)	35 (35.00%)	
Medical insurance			0.807
Free medical care	16 (6.08%)	7 (7.00%)	
Partly coverage	151 (57.41%)	60 (60.00%)	
Self-pay	96 (36.50%)	33 (33.00%)	
Economic status (RMB/month)			0.005*
<3000	104 (39.54%)	49 (49.00%)	
3000–6000	126 (47.91%)	30 (30.00%)	
>6000	33 (12.55%)	21 (21.00%)	
VA in better eyes	4.60 ± 0.58	4.33 ± 0.66	<0.001*
VA in worse eyes	3.42 ± 1.47	3.76 ± 0.92	0.945
Type of glaucoma			
PACG	104 (39.54%)	/	/
POAG	159 (60.46%)	/	/
Duration of diagnosis (months)	15.93 ± 26.48	19.94 ± 37.19	0.153
Religion belief			
Yes	23 (8.75%)	12 (12.00%)	0.348
No	240 (91.25%)	88 (88.00%)	
MUIS score	89.73 ± 10.26	82.92 ± 12.12	<0.001*
HADS score	11.56 ± 7.18	8.10 ± 5.92	<0.001*
Anxiety			0.024*
≥8	78 (29.66%)	18 (18.00%)	
<8	185 (70.34%)	82 (82.00%)	
Depression			0.055
≥8	73 (27.76%)	18 (18.00%)	
<8	190 (72.24%)	82 (82.00%)	
HADS-A	5.99 ± 3.94	4.41 ± 3.18	0.001*
HADS-D	5.59 ± 3.90	3.69 ± 3.15	<0.001*

VA: visual acuity, PACG: primary angle-closure glaucoma, POAG: primary open-angle glaucoma, MUIS: Mishel Uncertainty in Illness Scale, HADS: Hospital Anxiety and Depression Scale, HADS-A: HADS-anxiety, HADS-D: HADS- depression, ^*^P < 0.05.

### Comparison between scores of MUIS and HADS in glaucoma and cataract groups

The mean scores of MUIS (89.73 ± 10.26 vs 82.92 ± 12.12; p < 0.001) and HADS (11.56 ± 7.18 vs 8.10 ± 5.92; p < 0.001) were significantly higher in the glaucoma group than in the cataract group. The rates of anxiety were 29.66% (78/263) and 18.00% (18/100) in the glaucoma and cataract group, respectively (p = 0.024). And the rates of depression were 27.76% (78/263) and 18.00% (18/100), respectively (p = 0.055). Glaucoma patients are more likely to suffer from anxiety than cataract patients. The rates of depression for the two diseases showed no significant difference (Table 1).

### Impact factors associated with illness uncertainty, anxiety and depression in glaucoma and cataract groups

To identify impact factors for illness uncertainty, anxiety and depression in the two diseases, we conducted univariate logistic analysis. For glaucoma patients, high illness uncertainty was significantly correlated with HADS score (OR = 1.075, p = 0.003) and sub-score of HADS-A (OR = 1.118, p = 0.008) and HADS-D (OR = 1.104, p = 0.019). Anxiety was significantly associated with education level (p = 0.049), VA in the worse eye (OR = 0.772, p = 0.004), MUIS score (OR = 1.032, p = 0.022) and mean deviation (MD) in the better eye (p = 0.027). Depression was significantly associated with education level (p = 0.016) and MUIS score (OR = 1.028, p = 0.045). When the p value was set to <0.1, income per month (p = 0.088) and MD in the better eye (p = 0.052,) were observed to be impact factors for high illness uncertainty. In addition, economic status (p = 0.057) and VA in the better eye (OR = 0.654, p = 0.055) were impact factors for anxiety. Sex (OR = 1.687, p = 0.059) and VA in the worse eye (OR = 0.850, p = 0.074) were impact factors for depression. The details were shown in Table 2.

**Table 2:** Factors associated with high MUIS, anxiety and depression for glaucoma patients by univariate logistic analysis.

Variables	High MUIS				Anxiety				Depression			
	OR	95% CI		*P*	OR	95% CI		*P*	OR	95% CI		*P*
Age	1.006	0.986	1.026	0.548	0.993	0.974	1.011	0.433	1.001	0.982	1.021	0.886
Sex	1.174	0.666	2.070	0.578	1.294	0.761	2.198	0.341	1.687	0.979	2.908	0.059
Education level				0.158				0.049*				0.016*
College or higher	1				1				1			
High school	1.437	0.674	3.065	0.348	1.541	0.648	3.668	0.328	0.849	0.370	1.950	0.700
Primary or below	2.194	0.966	4.986	0.061	2.621	1.095	6.270	0.030	1.973	0.869	4.479	0.104
Medical insurance				0.762				0.332				0.671
Free medical care	1				1				1			
Partly coverage	1.364	0.430	4.322	0.598	3.030	0.647	14.194	0.159	1.610	0.424	6.108	0.484
Self-pay	1.506	0.490	4.630	0.474	3.163	0.691	14.480	0.138	1.782	0.484	6.562	0.385
Economic status (RMB/month)				0.088				0.057				0.225
<3000	2.569	1.102	5.990	0.029	2.700	1.024	7.120	0.045	2.489	0.881	7.032	0.085
3000–6000	2.080	0.926	4.674	0.076	1.597	0.606	4.211	0.344	2.240	0.802	6.256	0.124
>6000	1				1				1			
VA in better eyes	0.981	0.603	1.597	0.939	0.654	0.424	1.010	0.055	0.716	0.463	1.107	0.133
VA in worse eyes	0.937	0.769	1.141	0.515	0.772	0.647	0.920	0.004*	0.850	0.711	1.016	0.074
Type of glaucoma	0.794	0.449	1.406	0.429	0.868	0.503	1.497	0.611	0.673	0.382	1.187	0.172
Duration of diagnosis (months)	1.006	0.994	1.019	0.312	0.996	0.986	1.007	0.493	0.999	0.988	1.009	0.783
Religion belief	2.272	0.652	7.912	0.197	0.635	0.227	1.777	0.387	0.703	0.251	1.968	0.502
MD in better eye				0.052				0.027*				0.189
<12	1				1				1			
12–24	0.340	0.142	0.812	0.015	1.516	0.577	3.987	0.399	0.574	0.184	1.789	0.338
>24	0.832	0.321	2.156	0.705	3.292	1.377	7.872	0.007	1.857	0.773	4.460	0.166
MD in worse eye				0.520				0.387				0.839
<12	1				1				1			
12–24	0.604	0.254	1.435	0.253	1.182	0.460	3.040	0.729	1.117	0.465	2.682	0.805
>24	0.800	0.376	1.704	0.563	1.690	0.785	3.641	0.180	0.863	0.403	1.846	0.704
HADS score	1.075	1.025	1.128	0.003*								
Anxiety	2.446	1.014	1.199	4.988								
HADS-A score	1.118	1.029	1.214	0.008*								
Depressive	1.693	0.858	3.343	0.129								
HADS-D score	1.104	1.017	1.198	0.019*								
MUIS score					1.032	1.004	1.060	0.022*	1.028	1.001	1.056	0.045*

VA: visual acuity, MD: mean deviation, MUIS: Mishel Uncertainty in Illness Scale, HADS: Hospital Anxiety and Depression Scale, HADS-A:HADS-anxiety, HADS-D: HADS-depression, *P < 0.05.

For cataract patients, high illness uncertainty was significantly correlated with HADS score (OR = 1.091, p = 0.016), sub-score of HADS-A (OR = 1.220, p = 0.004) and depression (OR = 2.970, p = 0.047). Anxiety was significantly associated with VA in the better eye (OR = 0.236, p = 0.002) and VA in the worse eye (OR = 0.492, p = 0.007). Depression was only observed to be associated with high illness uncertainty (OR = 0.297, p = 0.047), as mentioned before. When the accepted p value was set to < 0.1, VA in the better eye (OR = 0.540, p = 0.075) and MUIS score (OR = 1.040, p = 0.082) were then found to be impact factors for depression. The details were shown in Table 3.

**Table 3:** Factors associated with high MUIS, anxiety and depression for cataract patients by univariate logistic analysis.

Variables	High MUIS				Anxiety				Depression			
	OR	95% CI		*P*	OR	95% CI		*P*	OR	95% CI		*P*
Age	0.983	0.944	1.024	0.418	1.015	0.962	1.072	0.577	1.039	0.981	1.101	0.186
Sex	0.741	0.336	1.633	0.457	2.000	0.685	5.839	0.205	0.498	0.175	1.414	0.190
Education level				0.143				0.269				0.430
College or higher	1				1				1			
High school	3.228	0.904	11.522	0.071	2.667	0.306	23.240	0.375	0.241	0.027	2.126	0.200
Primary or below	1.833	0.485	6.927	0.371	4.846	0.556	42.264	0.153	0.741	0.254	2.158	0.582
Medical insurance				0.242				0.816				0.751
Free medical care	1				1				1			
Partly coverage	4.421	0.477	40.980	0.191	1.071	0.105	10.914	0.954	2.885E8	0		0.999
Self-pay	6.000	0.681	52.900	6.000	1.500	0.165	13.667	0.719	4.468E8	0		0.999
Economic status (RMB/month)				0.106				0.255				0.540
<3000	3.068	1.020	9.231	0.046	3.081	0.625	15.198	0.167	2.436	0.485	12.237	0.280
3000–6000	1.667	0.504	5.510	0.405	1.462	0.242	8.820	0.679	2.375	0.430	13.128	0.321
>6000	1				1				1			
VA in better eyes	0.955	0.521	1.750	0.883	0.236	0.093	0.598	0.002*	0.540	0.274	1.064	0.075
VA in worse eyes	1.082	0.699	1.676	0.722	0.492	0.293	0.825	0.007*	0.670	0.416	1.081	0.101
Duration of diagnosis (months)	0.994	0.982	1.007	0.347	0.999	0.984	1.013	0.869	0.999	0.985	1.013	0.907
Religion belief	0.857	0.253	2.909	0.805	2.643	0.700	9.985	0.152	0.900	0.180	4.511	0.898
HADS score	1.091	1.017	1.172	0.016*								
Anxiety	1.679	0.601	4.690	0.323								
HADS-A score	1.220	1.065	1.399	0.004*								
Depression	2.970	1.014	8.699	0.047*								
HADS-D score	1.114	0.980	1.266	0.100								
MUIS score					0.997	0.956	1.040	0.904	1.040	0.995	1.088	0.082

VA: visual acuity, MD: mean deviation, MUIS: Mishel Uncertainty in Illness Scale, HADS: Hospital Anxiety and Depression Scale, HADS-A:HADS-anxiety, HADS-D: HADS-depression, *P < 0.05.

All variables that were significant at p < 0.1 in the univariate model were included into the multivariate stepwise logistic regression model to confirm the associations. The results showed that HADS scores (OR = 1.076, p = 0.013,), VA in the better eye (OR = 0.399, p = 0.020) and education level (p = 0.019) remained significantly associated with high illness uncertainty, anxiety and depression respectively for glaucoma patients (Table 4). Sub-score of HADS-A (OR = 1.220, p = 0.004), VA in the better eye (OR = 0.236, p = 0.002) and high illness uncertainty (OR = 3.025, p = 0.049) remained significantly associated with high illness uncertainty, anxiety and depression respectively for cataract patients (Table 5).

**Table 4:** Factors associated with High MUIS, anxiety and depression for glaucoma patients by multivariate logistic analysis.

Variables	Factors	EXP (B)[95% CI]	*P*
High MUIS	HADS score	1.076 [1.015, 1.139]	0.013*
Anxiety	VA in better eyes	0.399 [0.185, 0.863]	0.020*
Depression	Education level		0.019*
	College or higher	1	
	High school	0.832[0.360, 1.923]	
	Primary or below	1.924[0.842, 4.397]	

VA: visual acuity, HADS: Hospital Anxiety and Depression Scale, MUIS: Mishel Uncertainty in Illness Scale, *P < 0.05.

**Table 5:** Factors associated with MUIS, anxiety and depression for cataract patients by multivariate logistic analysis.

Variables	Factors	EXP (B)[95% CI]	*P*
High MUIS	HADS-A score	1.220 [1.065, 1.399]	0.004*
Anxiety	VA in better eyes	0.236 [0.093, 0.598]	0.002*
Depression	VA in better eyes	0.525 [0.263, 1.049]	0.068
	High MUIS score	3.025 [1.004, 9.116]	0.049*

VA: visual acuity, MUIS: Mishel Uncertainty in Illness Scale, HADS: Hospital Anxiety and Depression Scale, HADS-A:HADS-anxiety, *p < 0.05.

### Changes of illness uncertainty, anxiety and depression after surgery

We collected 25 glaucoma patients and 21 cataract patients who were evaluated with the same set of questionnaires before and after surgical procedures. Scores of MUIS and HADS both decreased after surgery, but the change of HADS score for glaucoma patients was not significant (MUIS:87.20 ± 11.12 vs 85.64 ± 11.91, p = 0.019; 79.24 ± 9.99 vs 76.14 ± 10.11, p = 0.011. HADS: 10.68 ± 7.52 vs 9.88 ± 8.50, p = 0.238; 6.62 ± 4.55 vs 5.24 ± 4.70, p = 0.009). The details were shown in Table 6.

**Table 6:** Score differences of MUIS, HADS, HADS-A, HADS-D in the two kinds of patients between before and after surgery.

Variables	Glaucoma			Cataract		
	Before	After	*p*	Before	After	*p*
MUIS	87.20 ± 11.12	85.64 ± 11.91	0.019*	79.24 ± 9.99	76.14 ± 10.11	0.011*
HADS	10.68 ± 7.52	9.88 ± 8.50	0.283	6.62 ± 4.55	5.24 ± 4.70	0.009*
HADS-A	5.92 ± 4.52	5.24 ± 4.65	0.200	3.52 ± 2.77	2.57 ± 2.96	0.018*
HADS-D	4.76 ± 3.38	4.64 ± 4.09	0.737	3.10 ± 2.72	2.67 ± 2.46	0.143

VA: visual acuity, MUIS: Mishel Uncertainty in Illness Scale, HADS: Hospital Anxiety and Depression Scale, HADS-A:HADS-anxiety, HADS-D: HADS-depression, *p < 0.05.

## Discussion

Over the past several decades, there has been increasing awareness concerning the importance of the mental health of patients with chronic disease including cataract and glaucoma. Addressing patients’ psychological disturbances is beneficial to therapy for physical disabilities. In this study, we evaluated the condition and relationship between illness uncertainty and two most common psychological disorders: anxiety and depression. We also identified risk factors associated with illness uncertainty and psychological disorders. Although anxiety and depression, as a common mood disorders in patients with chronic diseases, have been evaluated in glaucoma and cataract patients in several previous studies, to date illness uncertainty has not been assessed for the two diseases yet.

Illness uncertainty is always identified in chronic diseases due to such factors as long course of disease, lack of accurate information, complexity of the treatments, and loss of somatic functions and so on, so as glaucoma and cataract in our study^13,14^. We identified significantly higher illness uncertainty in glaucoma patients compared with cataract patients, probably because glaucoma tends to be associated with a younger onset age, more pain, more complications and more complex treatments, including both medications and surgeries^15,16^. The univariate logistic results showed us that illness uncertainty was positively associated with HADS score in both glaucoma patients and cataract patients. The multivariate logistic regression results confirmed that HADS score in glaucoma patients (OR = 1.076) and HADS-A score in cataract patients (OR = 1.220) were risk factors for high illness uncertainty, highlighting the association between illness uncertainty and psychological disturbances. In other diseases, such as cardiac patients, reducing illness uncertainty can help decrease a patient’s risk of crossing the borderline threshold of anxiety and depression^17^, which is consistent with our results.

Our study observed that the MUIS score of glaucoma and cataract patients were 89.73 ± 10.26 and 82.92 ± 12.12 respectively. As reported, Chinese men after cardiac catheterization had a MUIS score of 101.4 ± 11.49 in Hong Kong^18^, and Taiwanese lung cancer patients had a mean MUIS score of 79.46^19^. The mean MUIS score of Taiwanese breast cancer patients was 76.48. As for patients abroad, the uncertainty score of patients with chronic hepatitis C in the USA was 87.3 ± 17.6^14^, and prostate cancer patients had a uncertainty score of 64.1 ± 16.53^20^. Iran cancer patients had a MUIS score of 90.1 ± 16.8^21^. In our opinion, the differences in the MUIS score may be due to several reasons. Firstly, the studies adopted different versions of MUIS. Some studies chose MUIS of 33 items, and some chose the 25 items version. Secondly, different diseases have different treatments and prognosis and elicit different feelings. Thirdly, medical policy and culture in Chinese mainland differ from in other countries, so as in Hong Kong and Taiwan. And we should keep in mind that our study may suffer from a selection bias because our patients are all from one hospital in Guangzhou and cannot represent all glaucoma and cataract patients in China.

Based on our survey, the rates of anxiety and depression of glaucoma patients are 29.66% and 27.76%. And both rates are 18.00% of cataract patients. Reportedly, healthy people have an anxiety and depression rate of 7.0% and 5.2%. Previous studies showed an anxiety rate among glaucoma patients ranging from 13~64%^6,7,22,23^, and a depression rate ranging from 10.9~57.0%^6,22–24^.As for cataract patients in Russia, the rate of anxiety was reported to be 20%^25^, and the rate of depression ranged from 26~33.7%^25–27^, which is in keeping with our results.

VA in the better eye has been previously reported to impact mood status^28^. Our study also observed that VA in the better eye was the most important protective factor for anxiety in both glaucoma and cataract patients (OR = 0.399, p = 0.020 for glaucoma group, OR = 0.236, p = 0.002 for cataract group). Patients with better VA in their better eye were less likely to suffer from psychological disturbance indicating that attention should be paid to both eyes in the treatment of the affected eye. Education level was an impact factor for depression of glaucoma patients based on our study (p = 0.019). This is the first time to report that education level has a significant impact on depression. However we could not tell that if a high or low education level is helpful for preventing the occurrence of depression. Min Zhu had reported that lower educational attainment had worse vision-related quality of life^28^. That’s may because patients with lower education level have more difficulties to understand knowledge about the diseases, and their ability to deal with the situation is poor.

Controversies exist regarding the differences in psychological disorders between primary open angle glaucoma (POAG) and primary angle closure glaucoma (PACG). In our study, we failed to find a difference between POAG and PACG, similar to the conclusion of Nigel C.S^6^. A previous study reported that anxiety and depression are more prevalent in PACG than in POAG^29^. Larger-scale studies analyzing subgroups glaucoma patients or meta-analyses are required for clarification.

Age and sex have also been identified as risk factors for anxiety and depression previously. As reported by Chuandi Zhou, a younger age and female gender were independent risk factors for anxiety^7^. In another study from Japan, a younger age was a risk factor for anxiety and an older age was a risk factor for depression^8^, likely because younger patients have longer lifespan, and concern more about their remaining visual acuity. Unfortunately, age showed no correlation with either anxiety or depression in our study. Female glaucoma patients in Singapore were observed to be more vulnerable to depression than the males^6^ (p = 0.02). Female gender was shown to be an impact factor for the development of depression in the univariate model for glaucoma patients (OR = 1.687, p = 0.059) in our study. However, the correlation was not confirmed in the multivariate model.

Surgery can significantly alleviate illness uncertainty in the two diseases, but we observed that cataract patients were relieved of their psychological disorders after surgery, while glaucoma patients were not. Given that surgery is an effective treatment for both diseases, different outcomes suggest us that psychological distress may be intrinsic mechanisms of glaucoma. This hypothesis is similar with Bubella, R. M, who found that subjects with type A behavior experienced more frequent IOP fluctuations and were more sensitive to stress. Therefore, psychophysiological stress may play a role in the pathogenesis and evolution of OAG^30^. Further studies of molecular genetics and functional genetics are necessary to elucidate these connections.

It is meaningful to conduct studies to explore the emotional status of Chinese patients. As the largest developing country in the world, China has special conditions compared to other countries. We noticed that conclusions varied between our study and other studies. In addition to the differences in study design like adoption of different scales and the criteria for the patients and controls, we believe that the reasons below can further explain the differences. Firstly, doctors in China have heavy workloads and low salaries. Therefore, they experience low enthusiasm and have less time to communicate with patients^31^. Secondly, lack of health literacy among people in China has led to a poor understanding of long-term medication adherence, which is important for the process of therapy^32^. Patients want to be cured after one visit to the hospital, regardless of the complexity of their diseases. Thirdly, the investment in health-care is limited. China has 20% of the world’s population, but its health cost accounts for only 3% of the world’s total health-care burden. An insufficient health-care insurance system aggravates the economic burden for patients^31^. Fourthly, to increase the audience ratings, the media in China covers medical incidence incorrectly, leading to the demonization of doctors and nurses, and eliciting fear in patients when they get sick^33^.

There are some limitations in the present study that should be kept in mind. Firstly, the study scale is limited. Further studies with larger sample sizes will be necessary to examine these issues. Secondly, the variables included in this study are not enough. Other variables can influence the examined scales as well, such as the number of surgery patients has gone through^6^, the use of β-blocker eye drops^34^, and the progression rate of the disease^35^. Thirdly, the participants in our study were all from Zhongshan Ophthalmic Center. A collection bias may exist because Zhongshan Ophthalmic Center is the biggest and best eye hospital in China, and patients at this center have more complex conditions than in other eye hospitals. Larger-scale, multicenter researches are needed to assess patients’ actual illness uncertainty and psychological distress.

## Methods

### Subjects Enrollment Criteria

The study was a cross-sectional questionnaire-based study and was approved by the Ethical Review Committee of Zhongshan Ophthalmic Center. Informed consent was obtained from all participants in accordance with the principles embodied in the Declaration of Helsinki. The participants were recruited continuously between October 2013 and March 2016. All subjects were of the Chinese ethnic Han nationality. In this study, 263 patients with PACG or POAG and 100 patients with age-related cataract were recruited from the Departments of Glaucoma and Cataract at Zhongshan Ophthalmic Center. Among these subjects, 25 of the 263 glaucoma patients and 21 of the 100 cataract patients were evaluated with the same set of questionnaires before and after surgery.

All eligible participants had to be at least 18 years old and had to be capable of completing the questionnaires. In the glaucoma group, the participants had an established diagnosis of primary glaucoma in one or both eyes from a glaucoma ophthalmologist (Dr. M.K. LIN). Glaucoma was diagnosed on the basis of characteristic optic disc changes and characteristic visual field defects demonstrated on the Humphrey Visual Field Analyzer (HFA, Humphrey Instruments Inc., Allergan Humphrey, San Leandro, CA), with or without elevated intraocular pressure. Gonioscopy was performed to distinguish the subgroups of PACG and POAG. Patients with age related cataract were also enrolled. Cataracts were identified by slit-lamp examination with a portable slit lamp (model XL-1, Shin-Nippon, Japan). Participants were excluded from the study if (1) any eye disease was found rather than glaucoma or cataract (e.g., diabetic retinopathy, age-related macular degeneration); (2) patients with significant cataract badly affecting visual acuity in the glaucoma group; (3) patients who were diagnosis with other kind of glaucoma, such as exfoliation glaucoma or secondary glaucoma; (4) patients who had coexisting physical and psychiatric disorders like schizophrenia which has been reported to have a significant effect on patients’ psychological disturbance^36^.

### Data Collection

Before completing the questionnaires, all subjects were interviewed face-to-face, by well-trained nurses in their wards between 3:00 p.m.–5:00 p.m. each day to obtain demographic information. Basic information such as education level, religious faith, medical insurance and family income were recorded. Disease information such as diagnosis, visual acuity, the time interval from diagnosis to the interview and MD of HFA30-2 were also recorded. VA was determined by the Snellen visual acuity test chart at a distance of 5 m. If a patient could not recognize the largest optotype, then the distance would be reduced until he or she could read it. For patients who could not decipher the largest optotype at any distance, VA was tested in the order of finger counting, hand movement, and then light perception. VA was recorded as 5 minus logMAR. For illiterate patients and patients who could not read due to poor visual acuity, one specified nurse read the items to them in standard Chinese with the same statement for each item.

### Uncertainty Scale Measurement

The MUIS was used in this study. It is a 28-item checklist that measures uncertainty and ambiguity in terms of symptoms, diagnoses, treatments, relationships with caregivers, and planning for the future. It assesses four dimensions (ambiguity, complexity, inconsistency and unpredictability) on a 5-point Likert scale (from 1- strongly disagree, to 5- strongly agree)^37^. The reliability of the MUIS has been determined as from adequate to excellent and demonstrates good validity, as well as the Chinese version^18,19^. Score of MUIS ranges from 28–140. A higher MUIS score indicates a higher level of uncertainty. We described MUIS score of ≥84 as high illness uncertainty group.

### Anxiety and Depression Scale Measurement

The self-rating HADS was used in this study. It is a 14-item checklist on a 4-point Likert scale (from 0- strongly disagree, to 3- strongly agree) with 7 the items to assess anxiety (HADS-A) and another 7 to evaluate depression (HADS-D)^38^. The summary score of HADS ranges from 0–64, and score ≥8 in HADS-A/D indicates possible anxiety/depression^39^. A higher summary score from a patients’ self-rating indicates more severe anxiety and depression. The reliability and validity of the Chinese version of the HADS has been reported before^40^.

### Statistical analysis

The data were analyzed using SPSS17.0 software. The data were given as mean ± SD. Chi square test and t test (for normally distributed data) were performed for categorical variables and continuous data respectively, to examine the differences of participants’ clinical characteristics. If the data were not normally distributed (measured by Shapiro-Wilk value), the non-parametric test was applied instead. A univariate logistic regression was conducted to identify the influential factors for high illness uncertainty, anxiety and depression. All variables that were significant at p < 0.1 in the univariate logistic analysis were included into the multivariate stepwise logistic regression models after controlling age and sex as confounding factors. Statistical significance was accepted at p < 0.05.
